# Genetic diversity, phylogeography, population structure, and demographic history of wild *Catla catla* at a transboundary scale across South Asia revealed by Mitochondrial COI sequences

**DOI:** 10.1371/journal.pone.0341820

**Published:** 2026-02-02

**Authors:** J. K. Owaresat, Diptta Dey, Md. Ahashan Habib Siam, Md. Ashraful Anam, Ammam Zonaed Siddiki

**Affiliations:** 1 Department of Zoology, University of Chittagong, Chattogram, Bangladesh; 2 Department of Pathology and Parasitology, Faculty of Veterinary Medicine, Chittagong Veterinary and Animal Sciences University, Chattogram, Bangladesh; Bangladesh Agricultural University, BANGLADESH

## Abstract

This study presents the first assessment of mitochondrial cytochrome c oxidase I (COI) sequences from multiple countries to evaluate the genetic diversity, phylogeographic relationships, population structure, and demographic history of wild *Catla catla* in South Asia. A total of 18 haplotypes, with moderate haplotype diversity (Hd = 0.599), low nucleotide diversity (π = 0.017), and limited mutational steps among most haplotypes, were identified after analyzing 133 COI sequences collected from Bangladesh, India, and Pakistan. The results revealed low genetic differentiation among all wild Catla samples, influenced by introgression from hatchery-reared fry and population bottlenecks. Phylogenetic analyses identified two distinct haplogroups for Pakistani populations, supporting the existence of divergent mitochondrial lineages. AMOVA test showed that most genetic variation occurred within populations (74.46.%) rather than among the seven river basin populations (25.54%). The high pairwise genetic distance (FST= 0.255), together with the presence of numerous population-specific haplotypes and low gene flow (Nm = 0.729), indicated significant population structure among these river populations. A positive Mantel test (r = 0.12) confirmed a significant increase in genetic divergence with increasing geographic distance. The neutrality test and mismatch distribution presented a contrasting demographic history. A significantly negative Fu’s Fs (Fu’s Fs = −24.431) pointed to recent population expansion, whereas a significant Harpending’s raggedness index (r = 0.009) and a multimodal mismatch distribution suggested long-term demographic substructure. These findings provide essential COI-based baseline genetic information for conserving the genetic integrity of the wild *Catla catla* and guiding sustainable transboundary fisheries management in South Asia.

## 1. Introduction

*Catla catla,* commonly known as Catla (Hamilton, 1822; Cypriniformes: Cyprinidae), is the second most important Indian major carp due to its rapid growth rate, high consumer demand, and contribution to regional food security in South Asia [1,2]. This wild species is indigenous to the Indo-Gangetic riverine system in South Asia, including major rivers of India, Pakistan, Bangladesh, Nepal, Bhutan, and Myanmar [3,4]. *C. catla* is also extensively cultured in freshwater monoculture and polyculture systems alongside *Labeo rohita* and *Cirrhinus mrigala* throughout South Asia. Consequently, hatchery-produced fingerlings now serve as the primary source of seed across this region [5,6]. Catla plays a vital role in global freshwater aquaculture and regional economies, accounting for approximately 10.5% of global production, with an estimated market value of around USD 5 billion in 2018 [4,7,8]. However, the long-term survival of wild *Catla catla* populations in South Asia is increasingly threatened by overharvesting, dam construction, habitat degradation, pollution, and the emergence of invasive species [4,9–11]. These stressors disrupt spawning, migration, and early life-history stages, ultimately reducing effective population size, genetic diversity, and adaptive capacity of wild *C. catla* in South Asian river systems [4,12]. In addition, the widespread use of hatchery-reared broodstock and the escape of cultured fry into natural river systems in South Asia may result in genetic introgression, further eroding the genetic distinctiveness of wild Catla populations [11,13,14]. Although wild *C. catla* is currently listed as Least Concern by the IUCN [15], evaluating the level of genetic variability and population structure in these populations remains critical for developing effective conservation strategies [16,17].

Previous genetic studies on wild *C. catla* in South Asia have largely been country-specific and primarily employed mtDNA cyt *b* markers [13,18,19], microsatellite markers [2,3,11], SNP [20], and random amplified polymorphic DNA (RAPD) [4,21]. To date, no study has assessed the genetic diversity of wild Catla populations using the mtDNA cytochrome oxidase subunit I (COI) gene, nor has any investigation evaluated these populations across their transboundary distribution in South Asia. The mtDNA COI marker is now widely recognized as a universal and effective DNA barcode for investigating genetic diversity, phylogeography, and population structure in fishes, owing to its maternal inheritance, rapid evolutionary rate, protein-coding variation, and lack of recombination [5,22–24]. Because country-specific studies cannot fully capture the genetic landscape of this widely distributed fish in South Asia and COI-based analyses are lacking, this study aims to assess genetic variation, phylogeographic relationships, population structure, and demographic history of wild Catla populations based on mtDNA COI sequences from Bangladesh, India, and Pakistan. For the first time, this study will provide COI-based baseline genetic information at a transboundary scale to support conservation strategies and sustainable management of wild Catla populations across South Asia.

## 2. Materials and methods

### 2.1. Sample collection

#### 2.1.1. Data retrieval and filtering.

Mitochondrial cytochrome c oxidase I (COI) is widely used for DNA barcoding in fishes and offers reliable insights into intraspecific genetic variation and population structure. Although mitochondrial cyt *b* and nuclear markers have been employed to assess the genetic diversity of wild Catla populations, the COI marker has not yet been applied to investigate the genetic diversity and demographic history of these populations across South Asia at a transboundary scale.

For this study, mtDNA COI gene sequences of wild *C. catla* were retrieved from NCBI GenBank. Sequences were included if they met these criteria: (i) confirmed as wild *C. catla* and annotated as COI, (ii) at least 500 bp in length to provide sufficient genetic information (iii) high-quality sequences with no ambiguous bases or gaps (iv) clearly documented geographic metadata within South Asia, and (v) Identical sequences from the same locality were removed to reduce redundancy. Based on these criteria, a total of 133 mtDNA COI sequences (589–703 bp) from seven river systems in Bangladesh, India, and Pakistan were analyzed (NCBI accession numbers and geographic locations are provided in S1 Table).

#### 2.1.2. Geographic and river-basin grouping.

The dataset of 133 COI sequences comprised 10 sequences from the Jamuna-Meghna river basin and Halda River in Bangladesh, 95 sequences from the Indus-Beas, Ganga, Mahanadi, and Godavari river basins in India, and 28 sequences from the Indus river basin in Pakistan (S1 Table).

### 2.2. Data analysis

#### 2.2.1. Genetic diversity and phylogeographic analyses.

Multiple sequence alignment of the 133 COI sequences was performed with MAFFT v7 [25]. Low-quality regions, ambiguous bases, and uneven sequence ends were manually trimmed in BioEdit v7.7.1 [26]. The final alignments were visually inspected in MEGA v12 [27] to verify correct alignment and the overall quality of the sequences. Haplotype-based genetic diversity indices were calculated with DnaSP v6.12.05 [28], and a median-joining haplotype network was constructed in PopART v1.7 [29] to visualize haplotype relationships and distributions. Geographic distribution maps of haplotypes were generated using the maps package in R [30], based on publicly available spatial data from the CIA World Data Bank II [31]. Phylogeographic relationships among haplotypes were inferred through the Maximum Likelihood (ML) method, with 1,000 bootstrap replicates implemented in RAxML-NG v1.0.3 [32]. The best-fit nucleotide substitution model (HKY + F + G) was selected via IQ-TREE v2.0.7 [33], and phylogeographic trees were visualized and annotated with iTOL v5 [34].

#### 2.2.2. Population structure and demographic history analyses.

Analysis of Molecular Variance (AMOVA) and pairwise FST estimates were performed to assess the population structure among the seven selected river basin populations in South Asia, with 10,000 permutations conducted in Arlequin v3.5.2.2 [35]. Demographic history was evaluated through neutrality tests (Tajima’s D and Fu’s Fs) and mismatch distribution analyses (the sum of squared deviations and Harpending’s raggedness index), with 10,000 simulations performed in Arlequin v3.5.2.2. Observed and expected mismatch distributions were visualized in DnaSP v6. A Mantel test was conducted to examine the correlation between pairwise genetic distances (Kimura 2-parameter model) and geographic distances (Haversine formula) with 1,000 permutations implemented via the ape and vegan packages in R [36].

## 3. Results

### 3.1. Genetic diversity of wild *Catla catla p*opulations across South Asia

**Fig 1:**
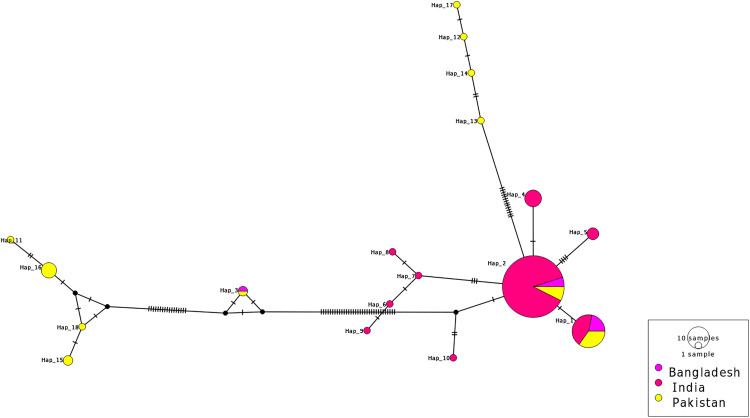
Median-joining network of mtDNA COI haplotypes in wild *Catla catla* populations across South Asia. Each circle represents a haplotype, with its size proportional to haplotype frequency. Colors indicate the country of origin, and the black lines on the branches denote the number of mutational changes between haplotypes.

Genetic diversity indices such as the number of haplotypes (h), the number of polymorphic sites (S), total number of mutations (Eta), haplotype diversity (Hd), nucleotide diversity (π), and the average number of nucleotide differences (k) were calculated based on 561 bp aligned COI sequences of 133 wild *Catla catla* samples across three South Asian countries. A total of 18 haplotypes were identified across the three countries: three types in Bangladesh (Hap_1-Hap_3), nine in India (Hap_1, Hap_2, Hap_4-Hap_10), and eleven in Pakistan (Hap_8, Hap_11-Hap_18). The geographic distribution of these 18 haplotypes is shown in S1 Fig (a,b), illustrating the frequency and regional distribution of each haplotype across the three countries. A median-joining haplotype network further depicted the shared haplotypes and the number of mutational steps among haplotypes across the three countries (Fig 1). This haplotype network displayed a complex distribution pattern where most haplotypes were separated by only a few mutational steps. Fifteen haplotypes were population-specific, whereas only three haplotypes (Hap_1, Hap_2, and Hap_3) were shared among all samples. Hap_1 and Hap_2 were identified as core haplotypes that were respectively shared by 23 and 81 Catla individuals across three countries (S2 Table). Overall values for the number of polymorphic sites (S), total number of mutations (Eta), haplotype diversity (Hd), nucleotide diversity (π), and the average number of nucleotide differences (k) were 75, 85, 0.599, 0.017, and 9.404, respectively, which indicate moderate haplotype diversity and low nucleotide divergence among wild Catla populations in South Asia (Table 1).

**Table 1:** Genetic diversity indices of wild *Catla catla* populations across South Asia.

n	S	ETA	h	Hd	Pi	k
133	75	85	18	0.599	0.017	9.404

(n = number of sequences, S = number of polymorphic sites, Eta = Total number of mutations, h = number of haplotypes, Hd = haplotype diversity, pi = nucleotide diversity, and k = average number of nucleotide differences).

### 3.2. Phylogeographic relationships among haplotypes of wild *Catla catla across* South Asia

**Fig 2:**
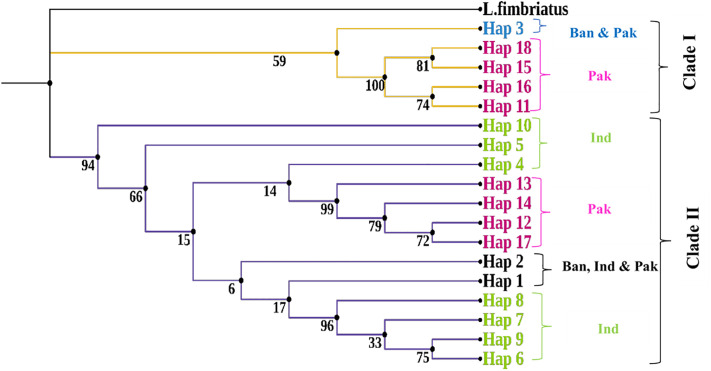
Maximum likelihood phylogeographic tree based on COI haplotypes of wild *Catla catla* populations across South Asia. *Labeo fimbriatus* was used as the outgroup. Country labels are as follows: Ban = Bangladesh, Ind = India, and Pak = Pakistan.

The maximum likelihood phylogeographic analysis resolved two major mitochondrial haplotypes clades among wild *Catla catla* samples from Bangladesh, India, and Pakistan (Fig 2). Clade I showed relatively low bootstrap support (59%), whereas Clade II was strongly supported (94%), suggesting uneven phylogeographic resolution. Despite this difference, both clades exhibited basal polytomies with mixed ancestry. Notably, haplotypes from the Pakistani Catla populations were distributed across two distinct clusters: Hap_11, Hap_15, Hap_16, and Hap_18 grouped within Clade I; and Hap_12, Hap_13, Hap_14, and Hap_17 clustered within Clade II. This phylogeographic pattern was further corroborated by the median-joining haplotype network (Fig 1).

### 3.3. Population differentiation of wild *Catla catla* populations across South Asia

**Fig 3:**
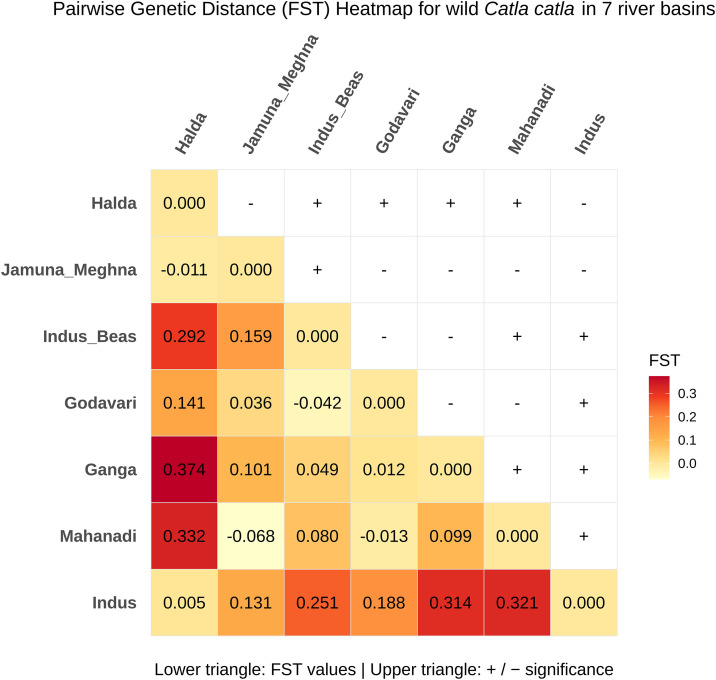
Pairwise genetic distance (FST) among Wild *Catla catla* populations across seven South Asian river basins. Values below the diagonal represent FST estimates, and values above the diagonal correspond to P-values. Significant differentiation (P ≤ 0.05) is indicated by the “+” symbol.

**Fig 4:**
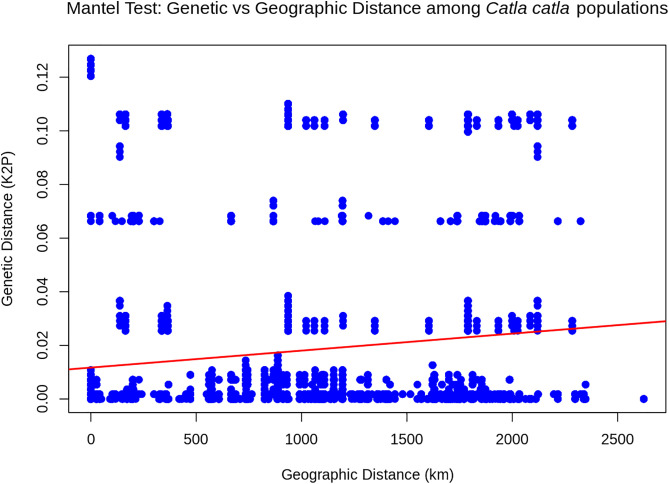
Mantel scatter plot for wild *Catla catla* populations across South Asia. The slope of the red regression line represents the overall trend of genetic divergence in relation to geographic distance.

The population structure among seven South Asian river basin populations of wild *Catla catla* was assessed by analysis of molecular variance (AMOVA) and pairwise genetic distance (FST). Significant AMOVA results showed that 25.54% (P < 0.001) of the genetic variation occurred among river populations, while 74.46% (P < 0.001) was within river populations (Table 2). Approximately half of the pairwise FST comparisons among river basin populations were not statistically significant (P < 0.05). The lowest significant FST (0.080) was observed between the Indus-Beas and Mahanadi river basin populations, while the highest FST (0.374) was found between populations from the Halda River and Ganga river basin (Fig 3). Overall population differentiation among the seven wild *Catla catla* populations was high (FST = 0.255, P < 0.001), and the estimated rate of gene flow was low (Nm = 0.729) (Table 2). A Mantel test revealed a significant positive correlation between pairwise genetic distances (Kimura 2-Parameter model) and geographic distances (Haversine formula) (r = 0.12, P < 0.05), with a weak but positive slope in the regression line (Table 3; Fig 4).

**Table 2:** AMOVA results for seven river basin populations of wild *Catla catla* across South Asia.

Sequence	Degrees of freedom (df)	Sum of squares	Variance component	Percentage of variation	Fixation index (FST)	P-value	Gene flow (Nm)
Among river populations	26	156.633	1.266	25.54%	0.255	0.000	0.729
Within river populations	126	464.983	3.690	74.46%			
Total	133	621.616	4.956	100%			

(Significant at P < 0.001)

**Table 3:** Mantel Test results for wild *Catla catla* populations across South Asia.

Statistic	Value	Significance (P-value)	Method	Permutation
Mantel Correlation (r)	0.12	0.003	Pearson	1000

(Significant at P < 0.05)

### 3.4. Demographic history analysis for wild *Catla catla* populations acrossSouth Asia

**Fig 5:**
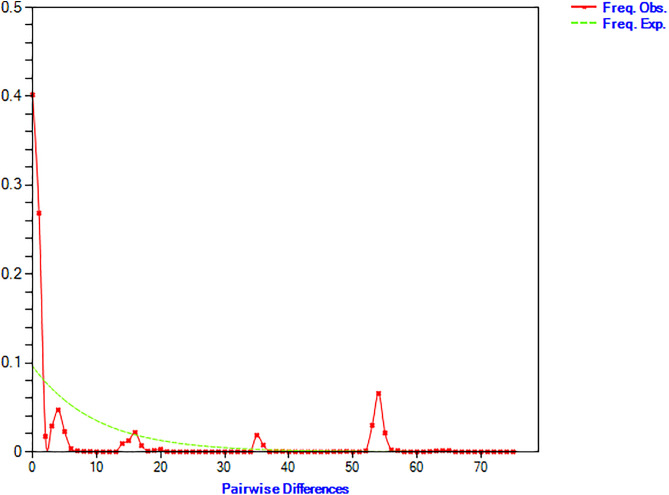
Mismatch distribution plot of wild *Catla catla* populations across South Asia. The X-axis represents pairwise nucleotide differences, and the Y-axis represents their frequency. The red line shows observed values, while the green line shows expected values under the sudden expansion model.

Neutrality tests (Tajima’s D and Fu’s Fs values) are commonly applied to infer evolutionary history and demographic events by detecting departures from the neutral mutation theory [37,38]. In this study, Tajima’s D value was not significant (−1.022, P > 0.05), whereas Fu’s Fs value was significantly negative (−24.431, P < 0.005), reflecting the rejection of neutral evolution (Table 4). Demographic history was further evaluated through the analysis of mismatch distribution parameters, such as the sum of squared deviations (SSD) [39] and Harpending’s raggedness index (r) [40,41] under the sudden expansion model. The sum of squared deviations value was non-significant (SSD = 0.036, P > 0.05), whereas the raggedness index (r) was statistically significant and low (r = 0.009, P < 0.05) (Table 4). Additionally, the pairwise mismatch distribution displayed a multimodal pattern for wild *Catla catla* populations in South Asia (Fig 5).

**Table 4:** Neutrality test and mismatch distribution parameters for wild *Catla catla* populations across South Asia.

n	Tajima’s D	P- value	FU’s Fs	P- value	SSD	P- value	r	P- value
133	−1.022	0.142	−24.431	0.000	0.036	1.000	0.009	0.000

(Significant at P < 0.05; n denotes the number of sequences, SSD = sum of squared deviations, r = Harpending’s raggedness index).

## 4. Discussion

Understanding the genetic variability and demographic history of wild Catla fish is fundamental for effective conservation and management of fisheries resources in South Asia. Haplotype diversity (Hd) and nucleotide diversity (π) are key indicators of genetic variation within and among populations, shaped by mutation, genetic drift, and gene flow [42]. The mitochondrial COI gene is particularly suitable for reconstructing haplotype relationships and detecting population structure in freshwater fishes due to its consistent phylogeographic resolution and high amplification success [43,44]. This study provides the first transboundary assessment of genetic variation, pylogeographic relationships, population-level differentiation and demographic history of wild *Catla catla* populations across South Asia based on 133 mtDNA COI sequences. A total of 18 haplotypes with moderate Haplotype diversity (Hd = 0.599) were identified across all samples (Table 1). The uneven distribution of these haplotypes, along with the presence of only two most frequent haplotypes (Hap_1 and Hap_2) among the three South Asian countries, supports the persistence of ancestral maternal lineages among all samples (S2 Table). In addition, low genetic differentiation among all 133 Catla individuals was supported by a low average number of nucleotide differences (k = 9.404) and minimal mutational distances among most haplotypes (Fig 1). Similar patterns of low genetic diversity were reported for other South Asian freshwater carps, such as *Catla catla* [20], *Labeo rohita* [5], and *Labeo gonius* [45]. The reduced genetic diversity of wild Catla populations may reflect founder effects or bottlenecks in South Asian river systems, caused by overharvesting, habitat degradation, the introduction of invasive species, and pollution [46–48]. Genetic introgression from hatchery-reared Catla, driven by extensive hatchery-based propagation and translocation of fingerlings [4,11,13], may also lead to this low genetic variation observed in wild Catla populations across South Asian rivers. Maximum likelihood phylogeographic analysis recovered two divergent mitochondrial haplogroups in two distinct clades for wild Catla populations from the Pakistani river basin (Fig 2). As Clade I had lower confidence (bootstrap 59%), it underscores the need for larger sample sizes to enhance this phylogeographic resolution. Nevertheless, the separation of cluster points to multiple maternal lineages or historically mixed sources of maternal ancestry within the Catla populations in the Indus river systems, Pakistan [49,50].

AMOVA results showed that most of the genetic variation occurred within the seven river basin populations of wild Catla rather than among populations (Table 2). This pattern was also reported in several previous studies on other freshwater carp species in this region [6,51]. Approximately 50% of the pairwise FST values between river basin populations were not statistically significant. It highlights the need to increase sample sizes for the Halda, Jamuna-Meghna, and Godavari river populations to improve FST estimates (Fig 3). However, the overall high pairwise genetic distance (FST) values and low gene flow (Nm) indicate substantial population differentiation among wild Catla populations from these seven river basins (Table 2). The predominance of fifteen population-specific haplotypes further supports the presence of population structuring (S2 Table; Fig 1). The positive correlation value from the Mantel test, along with a weak positive regression trend, suggests that geographic separation of seven river basins across South Asia contributes slightly to genetic differentiation among the seven river populations (Table 3; Fig 4). These findings likely reflect restricted maternal gene flow and local adaptation pressures resulting from limited geographical connectivity within river basins, habitat fragmentation due to dam construction, and heterogeneous environmental conditions [52–54].

Analyses of demographic history, including neutrality tests and mismatch distribution, presented partially contradictory insights. The significant negative Fu’s Fs value indicates an excess of rare haplotypes, supporting a recent population expansion of wild Catla populations across the South Asian region [52,55] (Table 4). In contrast, the significant Harpending’s raggedness index and the observed multimodal mismatch distribution (Table 4; Fig 5) suggest the presence of long-term population substructure within this region [41]. These contradictory results exhibit a complex demographic history that is likely influenced by both natural processes and strong anthropogenic pressures rather than a single demographic event. The non-significant Tajima’s D and SSD values may have contributed to this apparent contradiction if they had been statistically significant.

However, this first mtDNA COI-based assessment emphasizes the need for conservation of wild *Catla catla* populations across South Asia. Management strategies should prioritize maintaining natural connectivity among rivers and mitigating the impacts of overfishing, river dam constructions, pollution, and invasive species. Moreover, aquaculture practices should consider the genetic integrity of wild populations by restricting inter-basin translocations and hatchery releases, which are responsible for the genetic homogenization in wild Catla populations across this region.

## 5. Conclusion

The present study provides the first mitochondrial COI-based assessment of genetic diversity, pylogeographic relationships, population structure, and historical population dynamics of wild *Catla catla* across major river basins of South Asia. This analysis revealed low mitochondrial divergence, significant population differentiation, and evidence of a complex demographic history among wild Catla populations in the region. These findings suggest that the erosion of genetic diversity in wild *Catla catla* is likely associated with basin-specific anthropogenic pressures in South Asia. The results highlight the need for well-managed aquaculture practices to limit inter-basin translocations and hatchery releases that could compromise the genetic integrity of wild Catla populations.

The lack of mitochondrial COI data for wild Catla from other South Asian countries, such as Nepal, Bhutan, and Sri Lanka, as well as uneven geographic sampling of 133 COI sequences, represents a limitation of this study. Future studies incorporating additional mtDNA COI sequences from these countries, or employing nuclear markers (microsatellites, SNPs, or RAPD), mitochondrial markers (D-loop, cyt *b* regions), or whole mitochondrial genomes at a transboundary scale, would further refine complete understanding of the genetic structure, population differentiation, and demographic history of wild *C. catla* across various river basins in South Asia.

## Supporting information

S1 Table NCBI accession information for 133 mitochondrial COI sequences of wild *Catla catla* from the three South Asian regions. (DOCX)

S2 Table Relative frequencies of haplotypes among wild *Catla catla* populations across South Asia. (DOCX)

S1 Fig (a,b) Frequency and distribution of mtDNA COI haplotypes in wild Catla catla populations across South Asia. Pie sizes are proportional to the number of haplotypes, and slice sizes represent the relative frequency of each haplotype. Different colors denote distinct haplotypes. The map was generated using publicly available data from the CIA World DataBank II (1986) [30,31].
(DOCX)
