# Supplementary material for: Genetic diversity, phylogeography, population structure, and demographic history of wild Catla catla at a transboundary scale across South Asia revealed by Mitochondrial COI sequences
Source: PLoS One. 2026 Feb 2;21(2):e0341820. doi: 10.1371/journal.pone.0341820 (PMC12863562; doi:10.1371/journal.pone.0341820)
Supplement: S1 Table — (DOCX) [file pone.0341820.s001.docx]

**S1 Table. NCBI accession information for 133 mitochondrial COI sequences of Catla catla from the three South Asian regions.**

| **Accession Number** | ***Number*** | **Selected River Basin** | **Country** |
| --- | --- | --- | --- |
| >OR148096.1 to >OR148102.1  >OR148103.1;  >OR148104.1 to >OR148105.1;  >OR148106.1 to >OR148110.1;  >PP217418.1 to >PP217420.1.  (Himachal Pradesh) | 18 | Indus-Beas River system (32.18° N, 76.33° E). | India  (n=95) |
| >JX887594.1;>JX887592.1; >JX260838.1;>KX946601.1; >KX946602.1; >JX887593;  >KX163998.1;**>OP947652.1; MG736437.1.**  **(Maharashtra)** | 09 | Godavari River Basins  (19.59° N, 76.80° E). |  |
| >MK581182.1 to >MK581193.1; >HM026494.1;  >KF429914.1 to >KF429922.1;  >MK790055.1; >MK028751.1  >FJ459460.1 to ­>FJ459463.1;  >JQ801755.1; >JQ236669.1;  >JX983238.1; >JX983237.1  (Uttar Pradesh) | 32 | Ganga River Basin (**26.85° N, 80.95° E).** |  |
| >EU847510.1;  >KC757310.1 to >KC757327.1;  >GU195124.1;  >GU195050.1 to >GU195065.1.  **(Odisha)** | 36 | **Mahanadi River Basin**  **(20.70° N, 85.85° E).** |  |
| 1)  >OP575605.1 >OP575604.1;  >MK690395.1 ­to >MK690397.1;  >MN964128.1 to >MN964130.1  >MN355562.1 to >MN355565.1 (**Lahore)**  2)>KY228402.1 to >KY228414.1 (Gojra)  3) >MG229055.1; >MG229054.1;  >MG229056.1.  (**Muzaffarabad)** | 28 | Indus River Basin **(31.16° N, 72.68° E to 31.56° N, 74.35° E to 34.37° N, 73.47° E)** | Pakistan  (n=28) |
| 1) >MG969520.1;>MN259184.1 >LC823280.1  (Dhaka).  2) >MK572219.1; >MK416162.1 (Sherpur) | 05 | Jamuna-Meghna River Basin  (**23.81° N, 90.41° E to Near to 25.03° N, 90.01° E)** | **Bangladesh**  **(n=10)** |
| >MW509684.1 to >MW509688.1;  (Hald River) | 05 | Halda River (22.62° N, 91.75° E |  |

The dataset included 10 sequences from multiple sites in Dhaka (23.8103°N, 90.4125°E) and Sherpur (25.0220°N, 90.0011°E) under Jamuna-Meghna River Basin**,** and Chattogram (22.3569°N, 91.7832°E) under Halda River Bangladesh; 95 sequences from Himachal Pradesh (31.1048°N, 77.1734°E) under Indus-Beas River Basin**;** Uttar Pradesh (26.8467°N, 80.9462°E) and West Bengal (22.9868°N, 87.8550°E) under **Ganga River Basin,** Odisha (20.9517°N, 85.0985°E) under Mahanadi River Basin and Maharashtra (19.7515°N, 75.7139°E) under **Godavari River Basin;** and 28 sequences from Lahore (31.5497°N, 74.3436°E), Gojra (30.8833°N, 72.6786°E) and Muzaffarabad (34.3697°N, 73.4710°E) under Indus River Basin in Pakistan. No COI sequences were publicly available from Nepal, Bhutan, or Myanmar.
