# Supplementary material for: Genetic diversity, phylogeography, population structure, and demographic history of wild Catla catla at a transboundary scale across South Asia revealed by Mitochondrial COI sequences
Source: PLoS One. 2026 Feb 2;21(2):e0341820. doi: 10.1371/journal.pone.0341820 (PMC12863562; doi:10.1371/journal.pone.0341820)
Supplement: S2 Table — (DOCX) [file pone.0341820.s002.docx]

**S2 Table. **Relative frequencies of haplotypes among wild** Catla catla **populations across South Asia.****

| **Haplotypes** | **Bangladesh** | **India** | **Pakistan** |
| --- | --- | --- | --- |
| Hap-1 | 5 | 10 | 8 |
| Hap-2 | 4 | 71 | 6 |
| Hap-3 | 1 | 0 | 1 |
| Hap-4 | 0 | 6 | 0 |
| Hap-5 | 0 | 3 | 0 |
| Hap-6 | 0 | 1 | 0 |
| Hap-7 | 0 | 1 | 0 |
| Hap-8 | 0 | 1 | 0 |
| Hap-9 | 0 | 1 | 0 |
| Hap-10 | 0 | 1 | 0 |
| Hap-11 | 0 | 0 | 1 |
| Hap-12 | 0 | 0 | 1 |
| Hap-13 | 0 | 0 | 1 |
| Hap-14 | 0 | 0 | 1 |
| Hap-15 | 0 | 0 | 2 |
| Hap-16 | 0 | 0 | 5 |
| Hap-17 | 0 | 0 | 1 |
| Hap-18 | 0 | 0 | 1 |
