# Supplementary material for: Genetic diversity, phylogeography, population structure, and demographic history of wild Catla catla at a transboundary scale across South Asia revealed by Mitochondrial COI sequences
Source: PLoS One. 2026 Feb 2;21(2):e0341820. doi: 10.1371/journal.pone.0341820 (PMC12863562; doi:10.1371/journal.pone.0341820)
Supplement: S1 Fig — Pie sizes are proportional to the number of haplotypes, and slice sizes represent the relative frequency of each haplotype. Different colors denote distinct haplotypes. The map was generated using publicly available data from the CIA World DataBank II (1986) [30,31]. (DOCX) [file pone.0341820.s003.docx]

**S1 Figure (a,b). Frequency and distribution of mtDNA COI haplotypes in wild Catla catla populations across South Asia. Pie  **sizes are proportional to the number of haplotypes,** and **slice sizes represent the relative frequency of each haplotype**. Different colors denote distinct haplotypes. The map was generated using publicly available data from the CIA World DataBank II (1986) [30, 31].**

|  |
| --- |
| a |

|  |
| --- |
| **b** |
